# Potential benefits of converting from short daily high-flux hemodialysis to short daily online hemodiafiltration: a pilot study

**DOI:** 10.1590/2175-8239-JBN-2025-0127en

**Published:** 2026-03-06

**Authors:** Natalia Correa Vieira Melo, Lucio Mauricio do Rego Monteiro Isoni, Rossana Estima Duarte Simões, Ludimila D’Avila e Silva Allemand, Daniel França Vasconcelos, Juliane Pena Lauar, Istenio Fernandes Pascoal

**Affiliations:** 1 Centro Brasiliense de Nefrologia & Diálise, Brasília, DF, Brazil.; 2 Universidade do Distrito Federal, Escola Superior de Ciências da Saúde, Departamento de Pós-Graduação, Brasília, DF, Brazil.; 3 Universidade de Brasília, Departamento de Cardiologia, Brasília, DF, Brazil.; 4 Cardiosul Clínica Cardiológica, Brasília, DF, Brazil.

**Keywords:** Renal Dialysis, Hemodiafiltration, Renal Insufficiency, Chronic, Anemia, Cholesterol, Arterial Pressure

## Abstract

**Introduction:**

High-volume, conventional thrice-weekly hemodiafiltration appears to provide better outcomes than conventional hemodialysis. We aimed to evaluate whether the benefits of performing hemodiafiltration can be combined with those of frequent hemodialysis.

**Methods:**

This was a single-center, longitudinal, A1-B-A2 design study that compared high-flux short daily hemodialysis (SDHD) with post-dilution online short daily hemodiafiltration (SDHDF).

**Results:**

Twelve patients completed the study. Myoglobin clearance was greater during SDHDF (45.95 ± 11.0 mL/min) than during SDHD1 (20.11 ± 5.2 mL/min, P < 0.01) and SDHD2 (21.39 ± 3.55 mL/min, P < 0.01). A slightly higher pre-dialysis mean arterial pressure was observed during SDHDF (102.51 ± 11.28 mmHg) compared with SDHD1 (95.5 ± 9.82 mmHg, P < 0.01) and SDHD2 (97.61 ± 11.19 mmHg, P = 0.03). We also detected an increase in pre-dialysis hemoglobin during SDHDF (12.39 ± 1.2 g/dL) compared to SDHD1 (11.30 ± 1.09 g/dL, P = 0.02) and SDHD2 (11.23 ± 1.84 g/dL, P < 0.01). HDL-cholesterol was significantly higher at the end of the SDHDF period (52.75 ± 15.8 mg/dL) compared to SDHD1 (47.00 ± 10.8 mg/dL, P = 0.01) and SDHD2 (43.75 ± 11.1 mg/dL, P < 0.01) periods.

**Conclusion:**

The effect of converting SDHD to SDHDF was slightly positive in our study. In the long term, short daily hemodiafiltration might have more benefits for the dialysis population. This can lead to the adoption of hemodiafiltration as the standard of care even in the frequent hemodialysis setting.

## Introduction

Despite improvements in chronic hemodialysis over the years, patient morbidity and mortality remain high. Therefore, it is necessary to enhance dialysis treatment further to attempt to reduce the negative outcomes of patients undergoing hemodialysis. In this regard, frequent hemodialysis sessions using a more physiological regimen have shown better outcomes compared with conventional thrice-weekly hemodialysis. Improvements in volume overload, left ventricular mass, phosphate balance, and mortality have been reported in patients undergoing frequent dialysis, particularly in high-risk populations^1^. These benefits apparently are sustained in the long term^2^. Furthermore, similar advantages have been observed after conversion from conventional thrice-weekly hemodiafiltration to short daily hemodiafiltration (SDHDF)^3^.

Hemodiafiltration enhances the solute-removal spectrum by combining diffusion and convection, thus promoting a superior middle-molecule clearance compared with that provided by hemodialysis, while maintaining diffusive clearance^4,5^. High-volume conventional thrice-weekly hemodiafiltration apparently improves outcomes compared with conventional hemodialysis^4^. These benefits include not only cardiovascular protective effects, but also improvements in inflammatory status, anemia and hyperparathyroidism control, hemodynamic stability, and mortality^6,7,8,9,10^.

Nevertheless, it is noteworthy that high-flux hemodialysis is not a pure diffusion dialysis method, but rather a type of low-volume hemodiafiltration. This is because the internal filtration within the high-flux dialyzer can add significant convection clearance to the dialysis^4,11,12^. Similarly, the ultrapure dialysis water can reduce inflammation and improve anemia control^13^.

To the best of our knowledge, no studies to date have compared real-life relevant features between SDHDF and short daily high-flux hemodialysis (SDHD). Hence, it remains unknown whether the benefits of performing high-volume hemodiafiltration can be combined to those of frequent high-flux hemodialysis using ultrapure dialysis water. To address this question, we compared the clinical, biochemical, echocardiographic, and quality-of-life features between patients undergoing dialysis who were treated with short daily high-flux hemodialysis or short daily online hemodiafiltration.

## Methods

### Study Design and Population

This was a single-center, longitudinal, prospective, non-randomized, A1-B-A2 design study that compared high-flux SDHD with post-dilution online SDHDF. Each patient served as their own control. All 15 clinically stable patients enrolled on a regular SDHD program at the North Unit of the *Centro Brasiliense de Nefrologia & Diálise* for at least 6 months were included. The exclusion criteria were a vascular access unable to provide a blood flow greater than 300 mL/min and patient refusal to participate in the study.

The study was approved by the ethics committee of *Fundação de Ensino e Pesquisa em Ciências da Saúde*/ FEPECS/ SES/ DF on August 24, 2019 (approval number was 3,530,236). All participants signed the written free and informed consent forms approved by the ethics committee. The recruitment period started on November 1 and ended on November 17, 2019. Data collection started on November 18, 2019, and ended on November 21, 2020.

The period of 6 months on a regular short daily hemodialysis regimen (six times per week, 105–150 min per treatment) before the start of the study was considered the baseline period (SDHD1). Baseline data were collected, after which the patients’ treatment was converted to post-dilution SDHDF (six times per week, 105–150 min per treatment, mean total convection volume of 63.57 ± 5.44 L per week) for 6 months (SDHDF). The same data were collected at the end of the period of 6 months on SDHDF. Subsequently, all patients were switched back to SDHD for another 6 months (six times per week, 105–150 min per treatment) (SDHD2), and the same data were collected at the end of this phase.

Throughout the study, the dialysis parameters were matched and set as follows: high-flux and high-efficiency polysulfone dialyzers (Braun Xevonta Hi 23^®^; K0A, 1900 mL/min; Kuf, 124 mL/h/mmHg; sieving coefficient of β2-microglobulin >0.8; sieving coefficient of albumin <0.001; gamma sterilization), blood-flow rate of 300–350 mL/min, dialysate flow rate of 500 mL/min, and a Ultrapure Water System (Aquaboss^®^ heat disinfection osmosis) Dialog+^®^ Hemodialysis System (B Braun). During SDHDF, the post-dilution replacement volume was set as to guarantee a minimum replacement volume of 9.0 L/session and a total convection volume of 57 to 75 L/week. The total convection volume is defined as the total ultrafiltered volume, the sum of the replacement volume, and the intradialytic weight loss^14^.

The prescribed anticoagulants were individualized, and three patients (25%) needed a slight increase in the heparin dose after starting hemodiafiltration (SDHDF). Total ultrafiltration was set according to interdialytic weight gain and dry weight prescription. Medications were altered as appropriate for each patient by the treating physicians.

### Sample Collection

Pre-dialysis and post-dialysis blood samples were collected at baseline (SDHD1) and at the last sessions of both SDHDF and SDHD2, always during a midweek session. Blood samples were obtained from the arterial tubing before the dialysis session each period. The post-dialysis blood collection was performed 20 s after blood flow reduction to 100 mL/min and dialysate flow cut-off.

### Solute Removal and Dialysis Adequacy

The removal of substances with varying molecular weights was evaluated at baseline (SDHD1) and at the last session of SDHDF and SDHD2. The pre- and post-dialysis plasma concentrations of urea (60 D), creatinine (113 D), β2-microglobulin (11,800 D), and myoglobin (17,000 D) were measured. Moreover, the percentage reduction ratio of these solutes was calculated. The final concentration of ß2-microglobulin and myoglobin was subsequently corrected for extracellular volume contraction (hemoconcentration) and compartmentalization effect (post-dialysis rebound), according to appropriate formulas^15,16,17^.

Solute effective clearance was calculated on pre- and post-dialysis serum sampling values (blood-based simple kinetic model, skm). The effective or body clearance (K) of different solutes from the blood was calculated according to a single-pool model based on a log reduction using the pre-dialysis (S_pre_) and equilibrated post-dialysis (_eq_S_post_) concentrations corrected for hemoconcentration and rebound, as follows:


(1) $$\text{K soluto}=\text{Ln}\left({\text{S}}_{\text{pre}}{\text{/}}_{\text{eq}}{\text{S}}_{\text{post}}\right)\;\times \text{V/tHD}$$

where V stands for the solute volume of distribution and tHD is the duration of the dialysis session^15,16^.

The solute distribution volume was calculated using the Chertow formula and patient characteristics^18^. Total body water was used for urea and creatinine assessment, whereas the extracellular volume was calculated as one third of the total body water for β2-microglobulin and myoglobin. The net ultrafiltration volume was calculated as the difference between the pre-dialysis and post-dialysis weight and was used to calculate the average distribution volume.

Kt/V for all solutes was calculated using a simplified kinetic model (skm) according to the following equation^15,16^:


(2) $$\text{Kt/V soluto}=\text{Ln}\left({\text{S}}_{\text{pre}}{\text{/}}_{\text{eq}}{\text{S}}_{\text{post}}\right)$$

The dialysis dose offered was calculated to assess dialysis adequacy based on conventional markers of urea kinetics. The single-pool Kt/V (spKt/V) and the equilibrated Kt/V (eKt/V) were calculated using the Daugirdas equations^19^, and the standard Kt/V (stdKt/V) was obtained using the Leypoldt equation^20^.

### Clinical and Biochemical Parameters

The pre-dialysis levels of hemoglobin, urea, creatinine, potassium, total CO_2_, calcium, phosphorus, ferritin, transferrin saturation rate, parathyroid hormone (PTH), total serum protein, albumin, total cholesterol, HDL, low-density lipoproteins (LDL), triglycerides, β2-microglobulin, myoglobin, C-reactive protein, homocysteine, brain natriuretic peptide (BNP), fibrinogen, and interleukin-6 were measured at baseline (SDHD1) and at the last sessions of SDHDF and SDHD2, always during a midweek session. Dry weight and the medications used to treat anemia and bone disease were recorded at baseline (SDHD1) and at the last sessions of SDHDF and SDHD2. The occurrence of infections, antibiotic use, and hospital admissions during the study period were also recorded.

The systemic arterial blood pressure was measured immediately before the start and after the end of the dialysis session using an automatic blood pressure device. Episodes of intradialytic hypotension were recorded. In addition, the patients’ interdialytic weight gain was measured in each session, and the antihypertensive medications used at the baseline and at the end of each study period were recorded. Cardiac function and left ventricular hypertrophy were assessed using two-dimensional Doppler echocardiography, which was performed at baseline (SDHD1) and at the end of the SDHDF and SDHD2 periods always by the same cardiologist after a midweek dialysis session.

### Symptoms and Quality of Life

The presence and intensity of symptoms was assessed at baseline (SDHD1) and at the end of the SDHDF and SDHD2 periods using the revised Edmonton Symptom Assessment System adapted for chronic kidney disease on dialysis (ESAS-r: Renal)^21^.

At the baseline (SDHD1) and at the end of the SDHDF and SDHD2 periods, quality of life was assessed using the Kidney Disease and Quality-of-Life Short-Form (KDQOL-SF^TM^) instrument, which is a specific tool that assesses the quality of life of patients with chronic kidney disease and can be applied to patients undergoing dialysis. It is a self-administered instrument, which was previously validated for use in Brazil, with 80 items divided into 19 scales and takes approximately 16 min to be answered^22,23^.

### Statistical Analysis

The data were expressed as absolute frequencies and percentages (qualitative variables) or mean ± standard deviation (quantitative variables). Analysis of variance with repeated measures correction was used to compare quantitative variables. This model is part of linear mixed-effects models and is used in data analyses in which the responses are grouped (more than one measure for the same individual) and the assumption of independence between observations in the same group is not met^24^. These models assume that their residuals have a normal distribution with a mean of 0 and a constant σ^2^ variance. When this assumption was violated (as verified using normality tests and appropriate graphs), transformations of the response variable were used or outlier were excluded. A post-hoc test based on orthogonal contrasts was employed for comparisons. The log-binomial regression model with a random effect was used to compare the length of hospital stay and antibiotic use (binary answer: yes or no), which allowed estimation of relative risk^25^.

The data analysis performed in this study was carried out using the SAS/STAT^®^ software of the SAS System for Windows, version 9.4. (2013; SAS Institute Inc., Cary, NC, USA). A significance level of 5% was adopted for all comparisons.

## Results

No patient was excluded based on study protocol. However, two patients were excluded because of frequent absences from treatment, whereas another patient declined to provide written consent in the first month of the study. Therefore, 12 patients completed the study protocol (Table 1). No significant difference was observed regarding pre-dialysis dry weight or net ultrafiltration in the comparison of hemodiafiltration (SDHDF) with the two hemodialysis periods (SDHD1 and SDHD2). Dialysis adequacy was also similar among the study periods as observed by standard KTV measured during SDHDF (3.12 ± 0.29) compared with SDHD1 (3.20 ± 0.26, P > 0.05) or SDHD2 (3.34 ± 0.31, P > 0.05). Similarly, there was no difference in hospital admissions, the incidence of infections, or antibiotic use between the three study periods. During the SDHDF period, the post-dilution medium total convection volume was 64.46 ± 3.60 L/week.

**Table 1:** Patient characteristics

Characteristic	
Age (years)	60.8 ± 15.4
Sex	
Female, n (%)	5 (41.7%)
Male, n (%)	7 (57.3%)
Cause of end-stage renal disease	
Diabetic nephropathy, n (%)	4 (33%)
Arterial hypertension, n (%)	3 (25%)
Obstructive uropathy, n (%)	3 (25%)
Chronic glomerulonephritis, n (%)	2 (%)
Vascular access	
Native arteriovenous fistula, n (%)	7 (58.3%)
Long-term hemodialysis catheters, n (%)	4 (33.3%)
Arteriovenous graft, n (%)	1 (8.33%)
Time on hemodialysis (months)	32.6 ± 19.3
Usual hemodialysis session duration (min)	117.8 ± 8.63

Note – Values are expressed as the mean ± standard deviation or quantity (n) and percentage, as appropriate.

### Solute Removal

**Figure 1:**
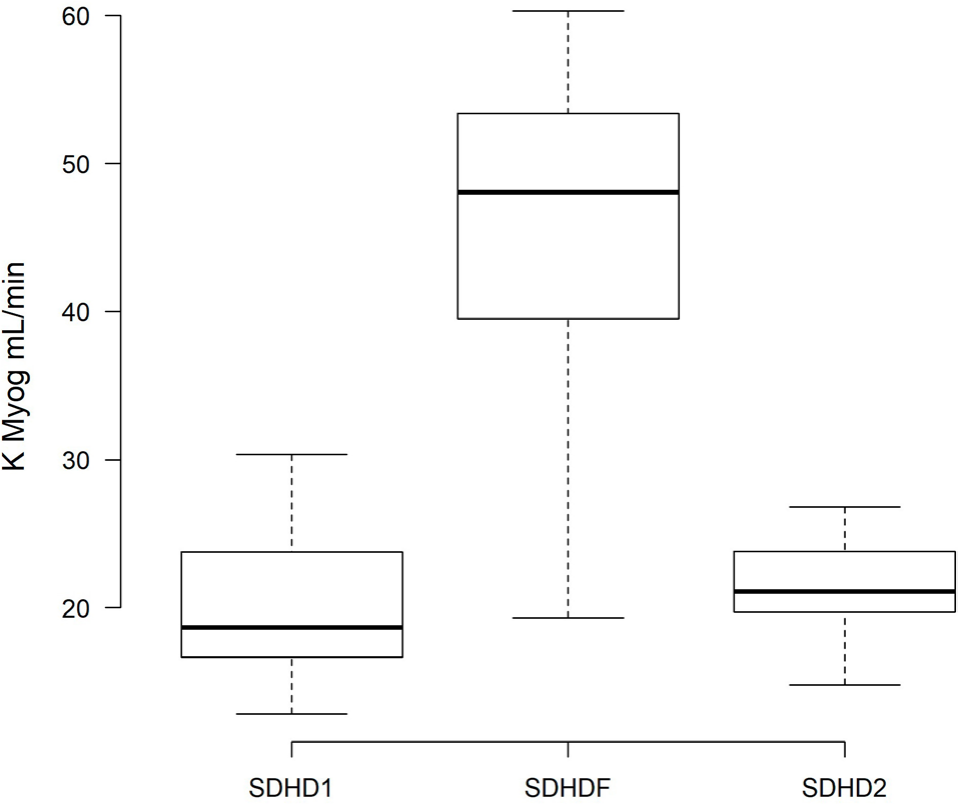
Myoglobin clearance (K Myog) at the basal hemodialysis period (SDHD1), after 6 months on hemodiafiltration (SDHDF), and after the second hemodialysis period (SDHD2).

There were no changes in the pre-dialysis solute concentration throughout the study (Table 2). However, solute kinetics measurements revealed a >100% greater myoglobin clearance during SDHDF (45.95 ± 11.0 mL/min) compared with SDHD1 (20.11 ± 5.2 mL/min, P < 0.01) and SDHD2 (21.39 ± 3.55 mL/min, P < 0.01) (Figure 1). Similarly, there was a slight increase in β2-microglobulin clearance in SDHDF (64.92 ± 10.4 mL/min) compared with SDHD1 (59.78 ± 17.0 mL/min, P < 0.01) and SDHD2 (53.01 ± 7.71 mL/min, P < 0.01). An unexplained slight improvement in urea and creatinine removal was observed in SDHD2 compared with SDHDF. No significant difference was observed in urea or creatinine clearance between SDHD1 and SDHDF (Table 2).

**Table 2:** Solute removal

	SDHD1	SDHDF	SDHD2	P-value (SDHD1 vs. SDHDF)	P-value (SDHDF vs. SDHD2)
Myoglobin					
Pre-myoglobin (ng/mL)	202.8 ± 101	197.5 ± 86.4	192.6 ± 88.8	ns	ns
K myoglobin (mL/min)	20.11 ± 5.20	45.95 ± 11.0	21.39 ± 3.55	P < 0.01	P < 0.01
β2-microglobulin					
Pre β2-microglobulin (mcg/mL)	21.27 ± 7.62	19.23 ± 10.5	20.24 ± 8.71	ns	ns
K β2-microglobulin (mL/min)	59.78 ± 17.0	64.92 ± 10.4	53.01 ± 7.71	P < 0.01	P < 0.01
Creatinine					
Pre-creatinine (mg/dL)	6.40 ± 2.62	6.19 ± 2.77	6.68 ± 3.06	ns	ns
K creatinine (mL/min)	184.5 ± 16.6	182.4 ± 23.9	199.2 ± 18.1	ns	P < 0.01
Urea					
Pre-urea (mg/dL)	86.67 ± 19.5	87.58 ± 26.6	89.42 ± 26.0	ns	ns
K urea (mL/min)	223.6 ± 23.5	224.4 ± 22.7	239.8 ± 21.8	ns	P < 0.01

Abbreviations – SDHD1: basal short daily hemodialysis; SDHDF: short daily hemodiafiltration; SDHD2: the second period of short daily hemodialysis; ns: not significant; Pre: pre-dialysis solute concentration; K: clearance.

Note – Values are expressed as the mean ± standard deviation.

### Cardiovascular Parameters

**Figure 2:**
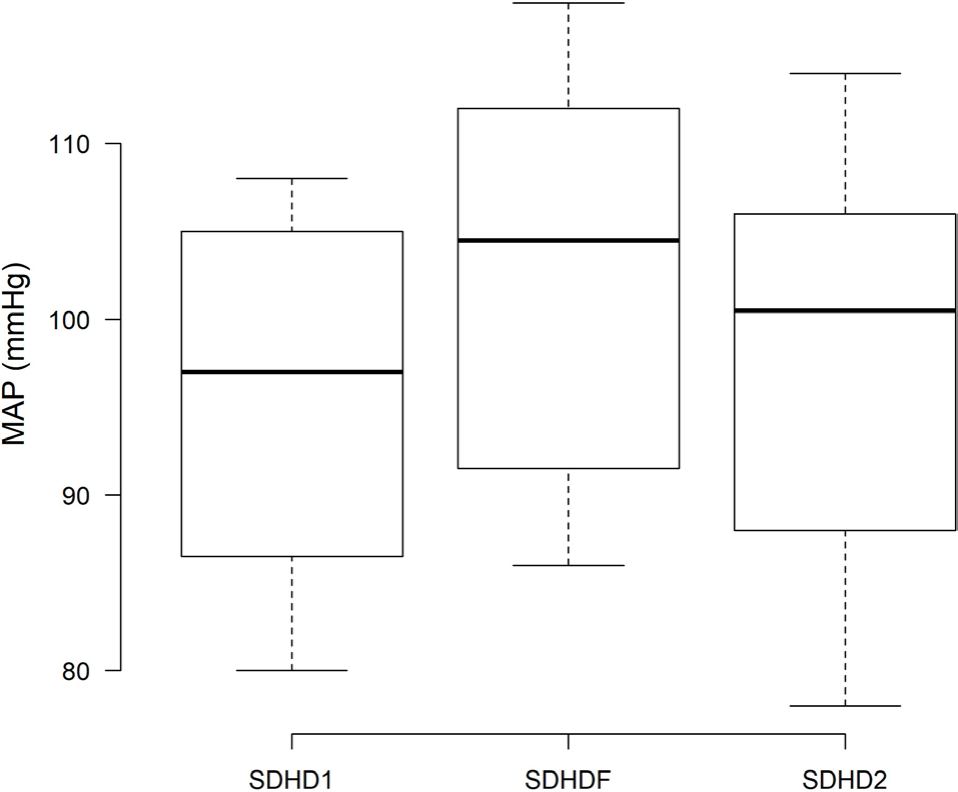
Pre-dialysis mean arterial pressure (MAP) at the basal hemodialysis period (SDHD1), after 6 months on hemodiafiltration (SDHDF), and after the second hemodialysis period (SDHD2).

A slightly higher pre-dialysis mean arterial pressure (MAP) was recorded during SDHDF (102.5 ± 11.3 mmHg) compared with SDHD1 (95.50 ± 9.82 mmHg, P < 0.01) and SDHD2 (97.61 ± 11.2 mmHg, P = 0.03) (Figure 2), with no significant differences detected in intradialytic blood pressure variability or antihypertensive drug doses. Finally, BNP and homocysteine levels remained stable throughout the study (Table 3).

**Table 3:** Cardiovascular, hematological, osteometabolic, and inflammatory parameters

	SDHD1	SDHDF	SDHD2	P-value (SDHD1 vs. SDHDF)	P-value (SDHDF vs. SDHD2)
BNP (pg/mL)	96.5 ± 198	113.4 ± 165	125.2 ± 196	ns	ns
Homocysteine (μmol/L)	17.51 ± 5.6	17.35 ± 4.9	19.36 ± 6.6	ns	ns
Hemoglobin (g/dL)	11.3 ± 1.09	12.39 ± 1.20	11.23 ± 1.84	P = 0.02	P < 0.01
Ferritin (ng/mL)	430.5 ± 356	466.13 ± 420	293.3 ± 293	ns	ns
TSAT (%)	20.74 ± 7.03	22.98 ± 9.26	19.28 ± 9.83	ns	ns
Potassium (mEq/L)	4.63 ± 0.76	4.66 ± 0.60	4.60 ± 0.65	ns	ns
Total CO_2_ (mmol/L)	24.78 ± 2.0	24.63 ± 1.5	23.59 ± 1.5	ns	ns
Calcium (mg/dL)	8.81 ± 0.53	8.78 ± 0.62	8.83 ± 0.48	ns	ns
Phosphorus (mg/dL)	4.78 ± 1.1	4.92 ± 1.3	5.08 ± 1.2	ns	ns
Serum PTH (pg/mL)	308.7 ± 201	339.3 ± 208	453.4 ± 230	ns	ns
Fibrinogen (mg/dL)	394.0 ± 104	349.7 ± 75.3	323.2 ± 70.8	ns	ns
C-reactive protein (mg/dL)	10.12 ± 10.4	8.72 ± 8.45	7.46 ± 8.46	ns	ns
Interleukin-6 (pg/mL)	8.63 ± 8.36	8.83 ± 4.13	7.84 ± 5.23	ns	ns

Abbreviations – SDHD1: basal short daily hemodialysis; SDHDF: short daily hemodiafiltration; SDHD2: the second period of short daily hemodialysis; ns: not significant; BNP: brain natriuretic peptide; TSAT: transferrin saturation rate; PTH: parathyroid hormone.

Note – Values are expressed as the mean ± standard deviation.

In turn, no significant changes in cardiac function or left ventricular hypertrophy were observed on echocardiography (Table 4). However, the longitudinal global strain was slightly worse at the end of SDHDF (−14.52% ± 1.96%) compare to that recorded at the end of SDHD2 (−16.63% ± 2.03%, P = 0.01). Such difference was not observed between SDHD1 (−15.56% ± 2.64%) and SDHDF (Table 4).

**Table 4:** Echocardiographic profile

	SDHD1	SDHDF	SDHD2	P-value (SDHD1 vs. SDHDF)	P-value (SDHDF vs. SDHD2)
Left ventricular mass (g)	175.8 ± 54.4	172.8 ± 43.2	177.0 ± 45.0	ns	ns
Left ventricular mass index (g/m^2^)	94.79 ± 28.7	92.83 ± 20.6	94.73 ± 21.3	ns	ns
Left atrial volume index (mL/m^2^)	19.94 ± 8.34	21.77 ± 10.4	27.21 ± 13.6	ns	ns
Left ventricular global longitudinal strain (%)	−15.56 ± 2.6	−14.52 ± 2.0	−16.63 ± 2.0	ns	P = 0.01
Intra-ventricular septum thickness (mm)	10.6 ± 2.2	11.1 ± 1.7	11.1 ± 1.4	ns	ns
Left atrial end-diastolic diameter (mm)	31.3 ± 4.8	32.1 ± 5.3	33.1 ± 6.5	ns	ns
Left ventricular ejection fraction (%)	67 ± 4.0	65 ± 5.0	65 ± 4.0	ns	ns
Left ventricular systolic diameter (mm)	28.9 ± 3.2	29.2 ± 2.9	28.8 ± 4.1	ns	ns
Left ventricular end-diastolic diameter (mm)	46.1 ± 5.1	45.4 ± 5.5	44.8 ± 6.3	ns	ns
Left ventricular posterior wall depth (mm)	10.5 ± 1.8	10.3 ± 1.3	10.9 ± 1.1	ns	ns

Abbreviations – SDHD1: basal short daily hemodialysis; SDHDF: short daily hemodiafiltration; SDHD2: the second period of short daily hemodialysis; ns: not significant.

Note – Values are expressed as the mean ± standard deviation.

### Hematological Parameters

**Figure 3:**
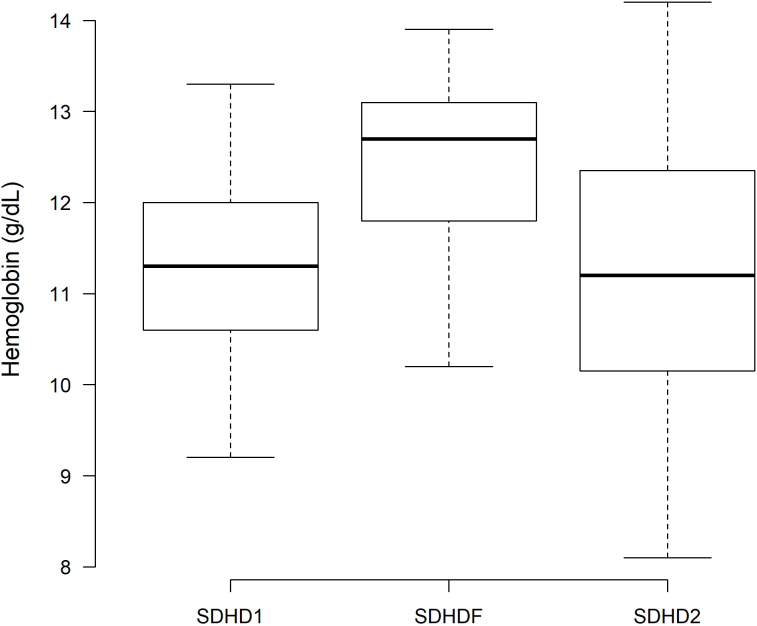
Pre-dialysis hemoglobin level at the basal hemodialysis period (SDHD1), after 6 months on hemodiafiltration (SDHDF), and after the second hemodialysis period (SDHD2).

There was an increase in pre-dialysis hemoglobin (Hb) levels after 6 months of SDHDF (12.39 ± 1.20 g/dL) compared with SDHD1 (11.30 ± 1.09 g/dL, P = 0.02), with a subsequent decrease recorded after SDHD2 (11.23 ± 1.84 g/dL, P < 0.01) (Figure 3). No significant differences were observed in the levels of the erythropoietin-stimulating agent, iron supplementation weekly doses, transferrin saturation rates, or ferritin levels (Table 3).

### Osteometabolic, Inflammatory, and Nutritional Parameters

The pre-dialysis osteometabolic (i.e., serum PTH, calcium, phosphorus, total CO_2_, potassium) and inflammatory (i.e., serum C-reactive protein, fibrinogen, and interleukin-6) parameters were not significantly different between SDHDF and SDHD1 or SDHD2 (Table 3).

**Figure 4:**
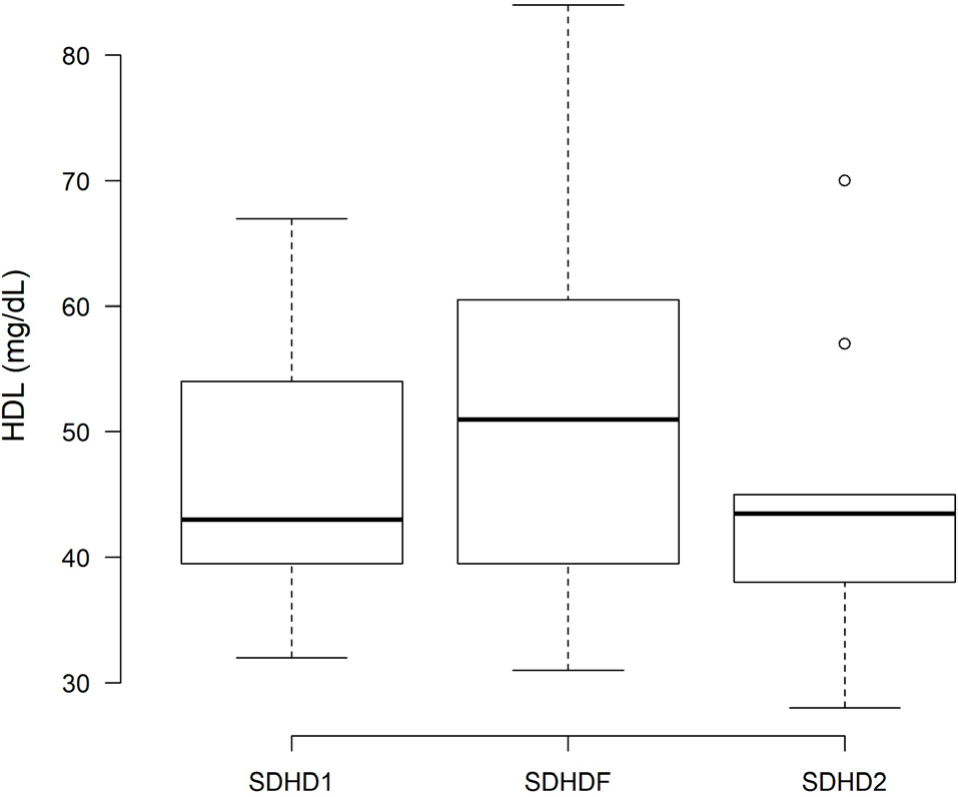
Pre-dialysis HDL-cholesterol level at the basal hemodialysis period (SDHD1), after 6 months on hemodiafiltration (SDHDF), and after the second hemodialysis period (SDHD2).

Regarding lipid profile, HDL-cholesterol was significantly higher at the end of the SDHDF period (52.75 ± 15.8 mg/dL) than that at the end of SDHD1 (47.00 ± 10.8 mg/dL, P = 0.01) and SDHD2 (43.75 ± 11.1 mg/dL, P < 0.01) (Figure 4). The levels of LDL-cholesterol and total cholesterol were similar between SDHD1 and SDHDF. However, both LDL and total cholesterol levels were lower at SDHD2 compared to SDHDF. No differences in albumin or total protein levels were observed between SDHDF and the two hemodialysis periods (Table 5).

**Table 5:** Nutritional parameters

	SDHD1	SDHDF	SDHD2	P-value (SDHD1 vs. SDHDF)	P-value (SDHDF vs. SDHD2)
Albumin (g/dL)	4.13 ± 0.41	4.03 ± 0.33	4.09 ± 0.28	ns	ns
Total protein (g/dL)	6.76 ± 0.43	6.56 ± 0.52	6.58 ± 0.46	ns	ns
Cholesterol (mg/dL)	180.7 ± 30.3	182.4 ± 33.6	159.1 ± 20.7	ns	P < 0.01
LDL (mg/dL)	101.6 ± 23.7	101.7 ± 24.4	85.75 ± 18.6	ns	P < 0.01
HDL (mg/dL)	47.00 ± 10.8	52.75 ± 15.8	43.75 ± 11.1	P = 0.01	P < 0.01
Triglycerides (mg/dL)	168.8 ± 87.4	175.0 ± 119	172.2 ± 102	ns	ns

Abbreviations – SDHD1: basal short daily hemodialysis; SDHDF: short daily hemodiafiltration; SDHD2: the second period of short daily hemodialysis; ns: not significant.

Note – Values are expressed as the mean ± standard deviation.

### Symptoms and Quality of Life

Regarding quality of life, SF-12 Physical Health Composite scores did not differ between SDHDF (40.02 ± 10.2) and SDHD1 (35.99 ± 10.3) or SDHD2 (39.54 ± 8.59). SF-12 Mental Health Composite scores also did not differ between SDHDF (51.43 ± 7.50) and SDHD1 (52.80 ± 13.5) or SDHD2 (49.11 ± 11.9). Moreover, no differences were detected in the total symptom distress score, as evaluated using ESAS-r: renal between SDHDF (19.25 ± 19.9) and SDHD1 (14.67 ± 15.5) or SDHD2 (24.08 ± 18.7).

## Discussion

This was the first study to compare relevant clinical features between short daily high-flux hemodialysis and short daily online hemodiafiltration. Slight benefits were observed after the conversion of the dialysis modality from SDHD to SDHDF. The benefits of hemodiafiltration were probably attenuated by the well-known benefits of short daily hemodialysis^1,2^.

A higher removal of medium-sized molecules, i.e., myoglobin and β2-microglobulin, was observed in short daily hemodiafiltration compared with short daily hemodialysis, especially of myoglobin, which has a larger molecular size than β2-microglobulin. This finding was similar to those of previous studies that compared high-flux hemodialysis with hemodiafiltration, and can be attributed to the higher convection clearance of online hemodiafiltration compared to hemodialysis^5,16,26,27^. However, no changes were observed in the pre-dialysis concentration of solutes with different sizes throughout the study. This result agrees with another study that compared thrice-weekly high-flux hemodialysis with thrice-weekly hemodiafiltration^28^. Nevertheless, a reduction in the pre-dialysis concentration of solutes with different sizes during hemodiafiltration compared with low-flux hemodialysis has been reported^8^, as well as with thrice-weekly hemodiafiltration compared with short daily hemodiafiltration^3^.

The present study found no changes in cardiac function or left ventricular hypertrophy evaluated by echocardiography, which was in contrast with the findings reported by other groups^6^. Throughout the study, the BNP and homocysteine levels did not vary. Although the pre-dialysis MAP levels were slightly higher during hemodiafiltration compared with the two hemodialysis periods, there were no differences in intradialytic blood pressure variability. Perhaps this occurred because the six-times-per-week regimen and its consequent interdialytic interval reduction are more important for cardiovascular stability and morbidity reduction than the dialysis modality itself^3,5,29,30^.

The pre-dialysis hemoglobin levels were higher at the end of the hemodiafiltration period compared with the two hemodialysis periods. No significant differences were observed in the weekly doses of erythropoietin-stimulating agent and iron supplementation, transferrin saturation rates, or ferritin levels. These findings agree with those of previous studies^7,31^ and are possibly related to a reduction in the resistance to erythropoiesis-stimulating agents observed in hemodiafiltration because of enhanced hepcidin clearance^32,33^.

Considering the lipid profile, HDL-cholesterol levels were significantly higher at the end of the SDHDF period compared with the two hemodialysis periods. This finding is in alignment with a prior comparison between hemodiafiltration and low-flux hemodialysis^8^. An enhancement in the removal of circulating inhibitors of lipoprotein lipase during hemodiafiltration may explain this improvement in lipid profile^8,34^.

No differences were observed in osteometabolic parameters between short daily hemodiafiltration and short daily hemodialysis. This finding is in contrast with the findings of several previous studies, which showed some improvement in phosphate control and/or pre-dialysis PTH levels after thrice-per-week hemodiafiltration compared with thrice-per-week hemodialysis^8,35,36^.

No changes in inflammation markers were observed throughout the study. This result differs from those of other studies, which reported an improvement in the inflammatory profile after hemodiafiltration compared with hemodialysis^37,38,39^. No differences were observed in the SF-12 Physical and Mental Health Composite scores among the treatment modalities assessed here, similar to a previously published study^40^. No differences were observed in the total symptom distress scores evaluated by ESAS-r: Renal. It is possible that not only the short daily schedule, but also the use of ultrapure water and highly biocompatible high-flux dialyzers during the entire study, diminished the potential advantages of hemodiafiltration itself regarding inflammation reduction and the improvement of quality of life and symptoms.

The main limitation of the present study was that it was performed at a single center using a small sample size. Multicentric studies with a larger number of patients are needed to confirm the findings of our study.

## Conclusion

In conclusion, our study revealed several important findings regarding the effects of short daily online hemodiafiltration compared to short daily high-flux hemodialysis. We observed a mild but notable increase in pre-dialysis mean arterial pressure, hemoglobin, and high-density lipoprotein cholesterol levels in patients undergoing hemodiafiltration. Additionally, there was a greater removal of medium-molecular-weight solutes during hemodiafiltration, which may contribute to improved patient outcomes. These results suggest that the biochemical and hemodynamic improvements associated with hemodiafiltration could be beneficial. In the long term, short daily hemodiafiltration might have more benefits for the dialysis population. Given these promising outcomes, there is a compelling case for the adoption of hemodiafiltration as the standard of care in frequent dialysis settings.

## Data Availability

The data that support the findings of this study are available from the corresponding author upon reasonable request.
