# Supplementary material for: Potential benefits of converting from short daily high-flux hemodialysis to short daily online hemodiafiltration: a pilot study
Source: J Bras Nefrol. 2026 Mar 6;48(1):e20250127. doi: 10.1590/2175-8239-JBN-2025-0127en (PMC12999184; doi:10.1590/2175-8239-JBN-2025-0127en)
Supplement: Translation

# Translation: Benefícios potenciais da conversão da hemodiálise curta diária de alto fluxo para a hemodiafiltração online curta diária: um estudo piloto

**DOI:** 10.1590/2175-8239-JBN-2025-0127pt

**Authors:** Natalia Correa Vieira Melo, Lucio Mauricio do Rego Monteiro Isoni, Rossana Estima Duarte Simões, Ludimila D’Avila e Silva Allemand, Daniel França Vasconcelos, Juliane Pena Lauar, Istenio Fernandes Pascoal

**Affiliations:** 1 Centro Brasiliense de Nefrologia & Diálise, Brasília, DF, Brasil.; 2 Universidade do Distrito Federal, Escola Superior de Ciências da Saúde, Departamento de Pós-Graduação, Brasília, DF, Brasil.; 3 Universidade de Brasília, Departamento de Cardiologia, Brasília, DF, Brasil.; 4 Cardiosul Clínica Cardiológica, Brasília, DF, Brasil.

**Keywords:** Diálise Renal, Hemodiafiltração, Insuficiência Renal Crônica, Anemia, Colesterol, Pressão Arterial

## Abstract

**Introdução:**

A hemodiafiltração convencional de alto volume, três vezes por semana, parece proporcionar desfechos superiores aos da hemodiálise convencional. Nosso objetivo foi avaliar se os benefícios da hemodiafiltração podem combinar-se aos da hemodiálise frequente.

**Métodos:**

Estudo longitudinal, de centro único, com desenho A1-B-A2, comparando hemodiálise curta diária (SDHD) de alto fluxo com hemodiafiltração online curta diária (SDHDF) pós-diluição.

**Resultados:**

Doze pacientes completaram o estudo. A depuração de mioglobina foi maior durante SDHDF (45,95 ± 11,0 mL/min) do que durante SDHD1 (20,11 ± 5,2 mL/min; P < 0,01) e SDHD2 (21,39 ± 3,55 mL/min; P < 0,01). Observou-se pressão arterial média pré-diálise ligeiramente mais elevada durante SDHDF (102,51 ± 11,28 mmHg) em comparação à SDHD1 (95,5 ± 9,82 mmHg; P < 0,01) e SDHD2 (97,61 ± 11,19 mmHg; P = 0,03). Também detectamos aumento na hemoglobina pré-diálise durante SDHDF (12,39 ± 1,2 g/dL) em comparação à SDHD1 (11,30 ± 1,09 g/dL; P = 0,02) e SDHD2 (11,23 ± 1,84 g/dL; P < 0,01). O colesterol HDL foi significativamente mais elevado no final do período da SDHDF (52,75 ± 15,8 mg/dL) em comparação aos períodos de SDHD1 (47,00 ± 10,8 mg/dL; P = 0,01) e SDHD2 (43,75 ± 11,1 mg/dL; P < 0,01).

**Conclusão:**

O efeito da conversão de SDHD para SDHDF foi ligeiramente positivo em nosso estudo. A longo prazo, a hemodiafiltração curta diária pode trazer mais benefícios para a população em diálise. Isso pode levar à adoção da hemodiafiltração como padrão de tratamento, mesmo em contextos de hemodiálise frequente.

## Introdução

Apesar dos avanços na hemodiálise crônica ao longo dos anos, a morbimortalidade dos pacientes permanece elevada. Portanto, é necessário aprimorar ainda mais o tratamento dialítico, na tentativa de reduzir os desfechos negativos de pacientes submetidos a essa modalidade. Nesse sentido, sessões frequentes de hemodiálise utilizando um regime mais fisiológico têm apresentado melhores desfechos em comparação à hemodiálise convencional, realizada três vezes por semana. Foram relatadas melhorias na sobrecarga volêmica, massa ventricular esquerda, equilíbrio de fosfato e mortalidade em pacientes submetidos à diálise frequente, particularmente em populações de alto risco^1^. Esses benefícios aparentemente se mantém a longo prazo. Além disso, vantagens semelhantes foram observadas após a conversão da hemodiafiltração convencional três vezes por semana, para a hemodiafiltração curta diária (SDHDF)^3^.

A hemodiafiltração melhora o espectro da remoção de solutos ao combinar difusão e convecção, promovendo, assim, uma depuração superior de moléculas médias em comparação à fornecida pela hemodiálise, ao mesmo tempo em que mantém a depuração difusiva^4,5^. A hemodiafiltração convencional de alto volume, realizada três vezes por semana, aparentemente melhora os desfechos quando comparada à hemodiálise convencional^4^. Esses benefícios incluem não apenas efeitos cardioprotetores, mas também melhorias no estado inflamatório, no controle da anemia e do hiperparatireoidismo, na estabilidade hemodinâmica e na mortalidade^6–10^.

No entanto, é importante notar que a hemodiálise de alto fluxo não constitui um método de diálise de difusão pura, mas sim um tipo de hemodiafiltração de baixo volume. Isso ocorre porque a filtração interna dentro do dialisador de alto fluxo pode adicionar uma depuração convectiva significativa à diálise^4,11,12^. De forma semelhante, o uso de água ultrapura para diálise pode reduzir a inflamação e aprimorar o controle da anemia^13^.

Até onde se tem conhecimento, nenhum estudo até o momento comparou características relevantes de vida real entre a SDHDF e a hemodiálise curta diária de alto fluxo (SDHD). Portanto, ainda não se sabe se os benefícios proporcionados pela hemodiafiltração de alto volume podem ser combinados aos da hemodiálise frequente de alto fluxo utilizando água ultrapura para diálise. Para abordar essa questão, comparamos as características clínicas, bioquímicas, ecocardiográficas e de qualidade de vida entre pacientes submetidos à diálise que foram tratados com hemodiálise curta diária de alto fluxo ou hemodiafiltração online curta diária.

## Métodos

### Desenho e População do Estudo

Trata-se de um estudo longitudinal, prospectivo, não randomizado, de centro único, com desenho A1-B-A2, que comparou a SDHD de alto fluxo com a SDHDF online pós-diluição. Cada paciente atuou como seu próprio controle. Foram incluídos todos os 15 pacientes clinicamente estáveis inscritos em um programa regular de SDHD na Unidade Norte do Centro Brasiliense de Nefrologia e Diálise por pelo menos 6 meses. Os critérios de exclusão foram: acesso vascular incapaz de fornecer fluxo sanguíneo superior a 300 mL/min e recusa do paciente em participar do estudo.

O estudo foi aprovado pelo Comitê de Ética em Pesquisa da Fundação de Ensino e Pesquisa em Ciências da Saúde/FEPECS/SES/DF em 24 de agosto de 2019 (número de aprovação 3.530.236). Todos os participantes assinaram os termos de consentimento livre e esclarecido aprovados pelo comitê de ética. O período de recrutamento teve início em 1º de novembro e encerrou-se em 17 de novembro de 2019. A coleta de dados ocorreu de 18 de novembro de 2019 a 21 de novembro de 2020.

O período de 6 meses em regime regular de hemodiálise curta diária (seis vezes por semana, 105–150 minutos por tratamento) antes do início do estudo foi considerado o período basal (SDHD1). Após a coleta dos dados basais, o tratamento dos pacientes foi convertido para SDHDF pós-diluição (seis vezes por semana, 105-150 minutos por tratamento, volume médio total de convecção de 63,57 ± 5,44 L por semana) por 6 meses (SDHDF). Os mesmos dados foram coletados ao final do período de 6 meses em SDHDF. Posteriormente, todos os pacientes foram novamente transferidos para SDHD por mais 6 meses (seis sessões por semana, 105–150 minutos por tratamento) (SDHD2), e os mesmos dados foram coletados ao término dessa fase.

Ao longo do estudo, os parâmetros de diálise foram ajustados e definidos da seguinte forma: dialisadores de polissulfona de alto fluxo e alta eficiência (*Braun Xevonta Hi 23* ^®^; K0A, 1900 mL/min; Kuf, 124 mL/h/mmHg; coeficiente de peneiramento da β2-microglobulina >0,8; coeficiente de peneiramento da albumina <0,001; esterilização por radiação gama), taxa de fluxo sanguíneo entre 300 e 350 mL/min, taxa de fluxo do dialisato de 500 mL/min e um Sistema de Hemodiálise *Dialog+*® (B Braun) com Sistema de Água Ultrapura (osmose com desinfecção térmica *Aquaboss* ^®^). Durante a SDHDF, o volume de reposição pós-diluição foi definido de modo a garantir um volume mínimo de reposição de 9,0 L/sessão e um volume total de convecção de 57 a 75 L/semana. O volume total de convecção é definido como o volume total ultrafiltrado, a soma do volume de reposição e a perda de peso intradiálitica^14^.

Os anticoagulantes prescritos foram individualizados, e três pacientes (25%) necessitaram de um leve aumento na dose de heparina após o início da hemodiafiltração (SDHDF). A ultrafiltração total foi definida de acordo com o ganho de peso interdialítico e a prescrição de peso seco. Os medicamentos foram alterados conforme apropriado para cada paciente, a critério dos médicos responsáveis pelo tratamento.

### Coleta de Amostras

Amostras sanguíneas pré e pós-diálise foram coletadas no período basal (SDHD1) e nas últimas sessões de SDHDF e SDHD2, sempre durante uma sessão realizada no meio da semana. As amostras sanguíneas foram obtidas da linha arterial, antes da sessão de diálise, para cada período. A coleta pós-diálise foi realizada 20 segundos após a redução do fluxo sanguíneo para 100 mL/min e a interrupção do fluxo de dialisato.

### Remoção de Solutos e Adequação da Diálise

A remoção de substâncias com pesos moleculares variáveis foi avaliada no período basal (SDHD1) e na última sessão de SDHDF e SDHD2. Foram medidas as concentrações plasmáticas pré e pós-diálise de ureia (60 D), creatinina (113 D), β2-microglobulina (11.800 D) e mioglobina (17.000 D). Além disso, foi calculada a taxa percentual de redução desses solutos. As concentrações finais de ß2-microglobulina e mioglobina foram posteriormente corrigidas para a contração do volume extracelular (hemoconcentração) e o efeito de compartimentação (rebote pós-diálise), de acordo com fórmulas apropriadas^15–17^.

A depuração efetiva dos solutos foi determinada com base nos valores séricos das amostras pré e pós-diálise, utilizando um modelo cinético simples baseado no sangue (*simple kinetic model*, SKM). A depuração efetiva ou corporal (K) de diferentes solutos do sangue foi calculada de acordo com um modelo de *pool* único, com base em uma redução logarítmica utilizando as concentrações pré-diálise (S_pre_) e pós-diálise equilibrada (_eq_S_post_) corrigidas para hemoconcentração e rebote, conforme descrito a seguir:


(1) $$\text{K soluto}=\text{Ln}\left({\text{S}}_{\text{pre}}{\text{/}}_{\text{eq}}{\text{S}}_{\text{post}}\right)\;\times \text{V/tHD}$$

onde V representa o volume de distribuição do soluto e tHD corresponde à duração da sessão de diálise^15,16^.

O volume de distribuição do soluto foi calculado por meio da fórmula de *Chertow* e das características dos pacientes^18^. A água corporal total foi utilizada para a avaliação de ureia e creatinina, enquanto o volume extracelular foi determinado como um terço da água corporal total para β2-microglobulina e mioglobina. O volume líquido de ultrafiltração foi calculado como a diferença entre o peso pré-diálise e pós-diálise e foi utilizado para o cálculo do volume médio de distribuição.

O Kt/V para todos os solutos foi obtido por meio de um modelo cinético simples (SKM), de acordo com a seguinte equação^15,16^:


(2) $$\text{Kt/V soluto}=\text{Ln}\left({\text{S}}_{\text{pre}}{\text{/}}_{\text{eq}}{\text{S}}_{\text{post}}\right)$$

A dose de diálise ofertada foi determinada para avaliar a adequação da diálise com base em marcadores convencionais de cinética da ureia. O Kt/V de *pool* único (spKt/V) e o Kt/V equilibrado (eKt/V) foram calculados utilizando as equações de *Daugirdas* ^19^, enquanto o Kt/V padrão (stdKt/V) foi obtido com a equação de *Leypoldt* ^20^.

### Parâmetros Clínicos e Bioquímicos

Os níveis pré-diálise de hemoglobina, ureia, creatinina, potássio, CO2 total, cálcio, fósforo, ferritina, índice de saturação da transferrina, paratormônio (PTH), proteínas séricas totais, albumina, colesterol total, HDL, lipoproteínas de baixa densidade (LDL), triglicerídeos, β2-microglobulina, mioglobina, proteína C reativa, homocisteína, peptídeo natriurético cerebral (BNP), fibrinogênio e interleucina-6 foram medidos no período basal (SDHD1) e nas últimas sessões de SDHDF e SDHD2, sempre durante uma sessão no meio da semana. O peso seco e os medicamentos utilizados para o tratamento da anemia e de doenças ósseas foram registrados no período basal (SDHD1) e nas últimas sessões de SDHDF e SDHD2. Também foram registradas a ocorrência de infecções, o uso de antibióticos e as internações hospitalares durante o período do estudo.

A pressão arterial sistêmica foi aferida imediatamente antes do início e após o término da sessão de diálise, utilizando um dispositivo automático de aferição da pressão arterial. Foram registrados os episódios de hipotensão intradialítica. Além disso, o ganho de peso interdialítico dos pacientes foi medido em cada sessão, e os medicamentos anti-hipertensivos utilizados no início e no final de cada período do estudo foram registrados. A função cardíaca e a hipertrofia ventricular esquerda foram avaliadas por meio de ecocardiograma bidimensional com Doppler, realizado no período basal (SDHD1) e no final dos períodos SDHDF e SDHD2, sempre pelo mesmo cardiologista, após uma sessão de diálise realizada no meio da semana.

### Sintomas e Qualidade de Vida

A presença e a intensidade dos sintomas foram avaliadas no período basal (SDHD1) e no final dos períodos SDHDF e SDHD2 utilizando o *Edmonton Symptom Assessment System* (Sistema de Avaliação de Sintomas de Edmonton) revisado e adaptado para doença renal crônica em diálise (ESAS-r: Renal)^21^.

No início do estudo (SDHD1) e no final dos períodos de SDHDF e SDHD2, a qualidade de vida foi avaliada com o instrumento *Kidney Disease and Quality-of-Life Short-Form* (KDQOL-SF^TM^), uma ferramenta específica que avalia a qualidade de vida de pacientes com doença renal crônica e pode ser aplicada àqueles em tratamento dialítico. Trata-se de um instrumento de autoaplicação, previamente validado para uso no Brasil, composto por 80 itens divididos em 19 domínios, com duração aproximada de 16 minutos para preenchimento^22,23^.

### Análise Estatística

Os dados foram expressos como frequências absolutas e porcentagens (variáveis qualitativas) ou média ± desvio padrão (variáveis quantitativas). A análise de variância com correção de medidas repetidas foi utilizada para comparar as variáveis quantitativas. Este modelo faz parte dos modelos lineares de efeitos mistos e é utilizado em análises de dados nas quais as respostas são agrupadas (mais de uma medida para o mesmo indivíduo) e a suposição de independência entre observações em um mesmo grupo não é atendida^24^. Esses modelos pressupõem que seus resíduos tenham uma distribuição normal com média igual a 0 e variância σ^2^ constante. Quando essa suposição foi violada (conforme verificado por meio de testes de normalidade e gráficos apropriados), foram utilizadas transformações da variável de resposta ou excluídos os valores discrepantes (*outliers*). Foi empregado um teste *post hoc* baseado em contrastes ortogonais para as comparações. O modelo de regressão log-binomial com efeito aleatório foi utilizado para comparar o tempo de internação e o uso de antibióticos (resposta binária: sim ou não), o que permitiu a estimativa do risco relativo^25^.

A análise dos dados deste estudo foi realizada utilizando o software SAS/STAT^®^ do *SAS System for Windows*, versão 9.4. (2013; *SAS Institute Inc.*, Cary, NC, EUA). Adotou-se um nível de significância de 5% para todas as comparações.

## Resultados

Nenhum paciente foi excluído com base no protocolo do estudo. No entanto, dois pacientes foram descartados devido a ausências frequentes nas sessões de tratamento, enquanto outro paciente se recusou a fornecer consentimento por escrito no primeiro mês do estudo. Dessa forma, 12 pacientes completaram o protocolo do estudo (Tabela 1). Não foram observadas diferenças significativas em relação ao peso seco pré-diálise ou à ultrafiltração líquida na comparação entre a hemodiafiltração (SDHDF) e os dois períodos de hemodiálise (SDHD1 e SDHD2). A adequação da diálise também foi semelhante entre os períodos do estudo, conforme observado pelos valores de Kt/V padrão obtidos durante a SDHDF (3,12 ± 0,29) em comparação com a SDHD1 (3,20 ± 0,26; P > 0,05) ou SDHD2 (3,34 ± 0,31; P > 0,05). Do mesmo modo, não houve diferença nas internações hospitalares, na incidência de infecções ou no uso de antibióticos entre os três períodos do estudo. Durante o período de SDHDF, o volume total de convecção do meio pós-diluição foi de 64,46 ± 3,60 L/semana.

**Tabela 1:** Características dos pacientes

Característica	
Idade (anos)	60,8 ± 15,4
Sexo	
Feminino, n (%)	5 (41,7%)
Masculino, n (%)	7 (57,3%)
Causa da doença renal em estágio terminal	
Nefropatia diabética, n (%)	4 (33%)
Hipertensão arterial, n (%)	3 (25%)
Uropatia obstrutiva, n (%)	3 (25%)
Glomerulonefrite crônica, n (%)	2 (%)
Acesso vascular	
Fístula arteriovenosa nativa, n (%)	7 (58,3%)
Cateteres para hemodiálise de longa permanência, n (%)	4 (33,3%)
Enxerto arteriovenoso, n (%)	1 (8,33%)
Tempo em hemodiálise (meses)	32,6 ± 19,3
Duração habitual da sessão de hemodiálise (min)	117,8 ± 8,63

Nota – Os valores são expressos como média ± desvio padrão ou quantidade (n) e porcentagem, conforme apropriado.

### Remoção de Solutos

Não houve alterações na concentração de solutos pré-diálise ao longo do estudo (Tabela 2). No entanto, as medições cinéticas do soluto revelaram uma depuração de mioglobina >100% maior durante a SDHDF (45,95 ± 11,0 mL/min) em comparação à SDHD1 (20,11 ± 5,2 mL/min; P < 0,01) e à SDHD2 (21,39 ± 3,55 mL/min; P < 0,01) (Figura 1). Da mesma forma, houve um discreto aumento na depuração da β2-microglobulina na SDHDF (64,92 ± 10,4 mL/min) em comparação com a SDHD1 (59,78 ± 17,0 mL/min; P < 0,01) e a SDHD2 (53,01 ± 7,71 mL/min; P < 0,01). Observou-se uma ligeira melhora inexplicável na remoção de ureia e creatinina na SDHD2 em comparação à SDHDF. Não houve diferença significativa na depuração de ureia ou creatinina entre a SDHD1 e a SDHDF (Tabela 2).

**Tabela 2:** Remoção de solutos

	SDHD1	SDHDF	SDHD2	Valor de p (SDHD1 vs. SDHDF)	Valor de p (SDHDF vs. SDHD2)
Mioglobina					
Pré-mioglobina (ng/mL)	202,8 ± 101	197,5 ± 86,4	192,6 ± 88,8	ns	ns
K Mioglobina (mL/min)	20,11 ± 5,20	45,95 ± 11,0	21,39 ± 3,55	P < 0,01	P < 0,01
β2-microglobulina					
Pré-β2-microglobulina (mcg/mL)	21,27 ± 7,62	19,23 ± 10,5	20,24 ± 8,71	ns	ns
K β2-microglobulina (mL/min)	59,78 ± 17,0	64,92 ± 10,4	53,01 ± 7,71	P < 0,01	P < 0,01
Creatinina					
Pré-creatinina (mg/dL)	6,40 ± 2,62	6,19 ± 2,77	6,68 ± 3,06	ns	ns
K creatinina (mL/min)	184,5 ± 16,6	182,4 ± 23,9	199,2 ± 18,1	ns	P < 0,01
Ureia					
Pré-ureia (mg/dL)	86,67 ± 19,5	87,58 ± 26,6	89,42 ± 26,0	ns	ns
K ureia (mL/min)	223,6 ± 23,5	224,4 ± 22,7	239,8 ± 21,8	ns	P < 0,01

Abreviaturas – SDHD1: hemodiálise curta diária basal; SDHDF: hemodiafiltração curta diária; SDHD2: segundo período de hemodiálise curta diária; ns: não significativo; Pré: concentração pré-diálise do soluto; K: depuração.

Nota – Os valores são expressos como média ± desvio padrão.

**Figura 1:** Depuração de mioglobina (K Miog) no período basal de hemodiálise (SDHD1), após 6 meses em hemodiafiltração (SDHDF) e após o segundo período de hemodiálise (SDHD2).

### Parâmetros Cardiovasculares

Foi registrada uma pressão arterial média (PAM) pré-diálise ligeiramente mais elevada durante a SDHDF (102,5 ± 11,3 mmHg) em comparação com a SDHD1 (95,50 ± 9,82 mmHg; P < 0,01) e a SDHD2 (97,61 ± 11,2 mmHg; P = 0,03) (Figura 2), sem diferenças significativas detectadas na variabilidade da pressão arterial intradialítica ou nas doses de medicamentos anti-hipertensivos. Por fim, os níveis de BNP e homocisteína permaneceram estáveis ao longo do estudo (Tabela 3).

**Figura 2:** Pressão arterial média (PAM) pré-diálise no período basal de hemodiálise (SDHD1), após 6 meses em hemodiafiltração (SDHDF) e após o segundo período de hemodiálise (SDHD2).

**Tabela 3:** Parâmetros cardiovasculares, hematológicos, osteometabólicos e inflamatórios

	SDHD1	SDHDF	SDHD2	Valor de p (SDHD1 vs. SDHDF)	Valor de p (SDHDF vs. SDHD2)
BNP (pg/mL)	96,5 ± 198	113,4 ± 165	125,2 ± 196	ns	ns
Homocisteína (μmol/L)	17,51 ± 5,6	17,35 ± 4,9	19,36 ± 6,6	ns	ns
Hemoglobina (g/dL)	11,3 ± 1,09	12,39 ± 1,20	11,23 ± 1,84	P = 0,02	P < 0,01
Ferritina (ng/mL)	430,5 ± 356	466,13 ± 420	293,3 ± 293	ns	ns
IST (%)	20,74 ± 7,03	22,98 ± 9,26	19,28 ± 9,83	ns	ns
Potássio (mEq/L)	4,63 ± 0,76	4,66 ± 0,60	4,60 ± 0,65	ns	ns
CO_2_ total (mmol/L)	24,78 ± 2,0	24,63 ± 1,5	23,59 ± 1,5	ns	ns
Cálcio (mg/dL)	8,81 ± 0,53	8,78 ± 0,62	8,83 ± 0,48	ns	ns
Fósforo (mg/dL)	4,78 ± 1,1	4,92 ± 1,3	5,08 ± 1,2	ns	ns
PTH sérico (pg/mL)	308,7 ± 201	339,3 ± 208	453,4 ± 230	ns	ns
Fibrinogênio (mg/dL)	394,0 ± 104	349,7 ± 75,3	323,2 ± 70,8	ns	ns
Proteína C reativa (mg/dL)	10,12 ± 10,4	8,72 ± 8,45	7,46 ± 8,46	ns	ns
Interleucina-6 (pg/mL)	8,63 ± 8,36	8,83 ± 4,13	7,84 ± 5,23	ns	ns

Abreviaturas – SDHD1: hemodiálise curta diária basal; SDHDF: hemodiafiltração curta diária; SDHD2: segundo período de hemodiálise curta diária; ns: não significativo; BNP: peptídeo natriurético cerebral; IST: índice de saturação da transferrina; PTH: paratormônio.

Nota – Os valores são expressos como média ± desvio padrão.

Por sua vez, não foram observadas alterações significativas na função cardíaca ou na hipertrofia ventricular esquerda no ecocardiograma (Tabela 4). No entanto, o *strain* global longitudinal apresentou leve piora ao final da SDHDF (−14,52% ± 1,96%) em comparação com o registrado ao término da SDHD2 (−16,63% ± 2,03%; P = 0,01). Essa diferença não foi observada entre SDHD1 (−15,56% ± 2,64%) e SDHDF (Tabela 4).

**Tabela 4:** Perfil ecocardiográfico

	SDHD1	SDHDF	SDHD2	Valor de p (SDHD1 vs. SDHDF)	Valor de p (SDHDF vs. SDHD2)
Massa ventricular esquerda (g)	175,8 ± 54,4	172,8 ± 43,2	177,0 ± 45,0	ns	ns
Índice de massa ventricular esquerda (g/m^2^)	94,79 ± 28,7	92,83 ± 20,6	94,73 ± 21,3	ns	ns
Índice de volume do átrio esquerdo (mL/m^2^)	19,94 ± 8,34	21,77 ± 10,4	27,21 ± 13,6	ns	ns
*Strain* longitudinal global do ventrículo esquerdo (%)	−15,56 ± 2,6	−14,52 ± 2,0	−16,63 ± 2,0	ns	P = 0,01
Espessura do septo interventricular (mm)	10,6 ± 2,2	11,1 ± 1,7	11,1 ± 1,4	ns	ns
Diâmetro diastólico final do átrio esquerdo (mm)	31,3 ± 4,8	32,1 ± 5,3	33,1 ± 6,5	ns	ns
Fração de ejeção do ventrículo esquerdo (%)	67 ± 4,0	65 ± 5,0	65 ± 4,0	ns	ns
Diâmetro sistólico do ventrículo esquerdo (mm)	28,9 ± 3,2	29,2 ± 2,9	28,8 ± 4,1	ns	ns
Diâmetro diastólico final do ventrículo esquerdo (mm)	46,1 ± 5,1	45,4 ± 5,5	44,8 ± 6,3	ns	ns
Profundidade da parede posterior do ventrículo esquerdo (mm)	10,5 ± 1,8	10,3 ± 1,3	10,9 ± 1,1	ns	ns

Abreviaturas – SDHD1: hemodiálise curta diária basal; SDHDF: hemodiafiltração curta diária; SDHD2: segundo período de hemodiálise curta diária; ns: não significativo.

Nota – Os valores são expressos como média ± desvio padrão.

### Parâmetros Hematológicos

Houve um aumento nos níveis de hemoglobina (Hb) pré-diálise após 6 meses de SDHDF (12,39 ± 1,20 g/dL) em comparação com a SDHD1 (11,30 ± 1,09 g/dL; P = 0,02), com uma redução subsequente registrada após a SDHD2 (11,23 ± 1,84 g/dL; P < 0,01) (Figura 3). Não foram observadas diferenças significativas nos níveis do agente estimulador da eritropoietina, nas doses semanais de suplementação de ferro, nos índices de saturação da transferrina ou nos níveis de ferritina (Tabela 3).

**Figura 3:** Nível de hemoglobina pré-diálise no período basal de hemodiálise (SDHD1), após 6 meses em hemodiafiltração (SDHDF) e após o segundo período de hemodiálise (SDHD2).

### Parâmetros Osteometabólicos, Inflamatórios e Nutricionais

Os parâmetros osteometabólicos pré-diálise (ou seja, PTH, cálcio, fósforo, CO_2_ total e potássio séricos) e inflamatórios (ou seja, proteína C reativa, fibrinogênio e interleucina-6 séricos) não apresentaram diferenças significativas entre SDHDF e SDHD1 ou SDHD2 (Tabela 3).

Em relação ao perfil lipídico, o colesterol HDL foi significativamente mais elevado no final do período de SDHDF (52,75 ± 15,8 mg/dL) quando comparado aos valores observados no final da SDHD1 (47,00 ± 10,8 mg/dL; P = 0,01) e da SDHD2 (43,75 ± 11,1 mg/dL; P < 0,01) (Figura 4). Os níveis de colesterol LDL e colesterol total foram semelhantes entre SDHD1 e SDHDF. No entanto, os níveis de colesterol LDL e colesterol total foram mais baixos na SDHD2 em comparação à SDHDF. Não foram observadas diferenças nos níveis de albumina ou proteína total entre a SDHDF e os dois períodos de hemodiálise (Tabela 5).

**Figura 4:** Nível de colesterol HDL pré-diálise no período basal de hemodiálise (SDHD1), após 6 meses em hemodiafiltração (SDHDF) e após o segundo período de hemodiálise (SDHD2).

**Tabela 5:** Parâmetros nutricionais

	SDHD1	SDHDF	SDHD2	Valor de p (SDHD1 vs. SDHDF)	Valor de p (SDHDF vs. SDHD2)
Albumina (g/dL)	4,13 ± 0,41	4,03 ± 0,33	4,09 ± 0,28	ns	ns
Proteína total (g/dL)	6,76 ± 0,43	6,56 ± 0,52	6,58 ± 0,46	ns	ns
Colesterol (mg/dL)	180,7 ± 30,3	182,4 ± 33,6	159,1 ± 20,7	ns	P < 0,01
LDL (mg/dL)	101,6 ± 23,7	101,7 ± 24,4	85,75 ± 18,6	ns	P < 0,01
HDL (mg/dL)	47,00 ± 10,8	52,75 ± 15,8	43,75 ± 11,1	P = 0,01	P < 0,01
Triglicérides (mg/dL)	168,8 ± 87,4	175,0 ± 119	172,2 ± 102	ns	ns

Abreviaturas – SDHD1: hemodiálise curta diária basal; SDHDF: hemodiafiltração curta diária; SDHD2: segundo período de hemodiálise curta diária; ns: não significativo.

Nota – Os valores são expressos como média ± desvio padrão.

### Sintomas e Qualidade de Vida

Quanto à qualidade de vida, os escores do componente físico do SF-12 não diferiram entre SDHDF (40,02 ± 10,2) e SDHD1 (35,99 ± 10,3) ou SDHD2 (39,54 ± 8,59). Da mesma forma, os escores do componente mental do SF-12 não diferiram entre SDHDF (51,43 ± 7,50) e SDHD1 (52,80 ± 13,5) ou SDHD2 (49,11 ± 11,9). Além disso, não foram detectadas diferenças no escore total de desconforto dos sintomas, avaliado por meio do ESAS-r: Renal entre SDHDF (19,25 ± 19,9) e SDHD1 (14,67 ± 15,5) ou SDHD2 (24,08 ± 18,7).

## Discussão

Este foi o primeiro estudo a comparar características clínicas relevantes entre a hemodiálise curta diária de alto fluxo e a hemodiafiltração online curta diária. Foram observados benefícios discretos após a conversão da modalidade de diálise de SDHD para SDHDF. Os benefícios da hemodiafiltração foram provavelmente atenuados pelos benefícios já conhecidos da hemodiálise diária de curta duração^1,2^.

Observou-se uma maior remoção de moléculas de tamanho médio, ou seja, mioglobina e β2-microglobulina, na hemodiafiltração curta diária em comparação à hemodiálise curta diária, especialmente da mioglobina, que apresenta um tamanho molecular superior ao da β2-microglobulina. Este achado foi semelhante ao de estudos anteriores que compararam a hemodiálise de alto fluxo com a hemodiafiltração e pode ser atribuído à maior depuração convectiva proporcionada pela hemodiafiltração online em relação à hemodiálise^5,16,26,27^. No entanto, não foram observadas alterações na concentração pré-diálise de solutos com diferentes tamanhos ao longo do estudo. Este resultado está de acordo com outro estudo que comparou a hemodiálise de alto fluxo três vezes por semana com a hemodiafiltração na mesma frequência^28^. No entanto, foi relatada uma redução na concentração pré-diálise de solutos com diferentes tamanhos durante a hemodiafiltração em comparação à hemodiálise de baixo fluxo^8^, bem como com a hemodiafiltração realizada três vezes por semana em comparação à hemodiálise curta diária^3^.

O presente estudo não encontrou alterações na função cardíaca ou hipertrofia ventricular esquerda avaliadas por ecocardiograma, em contraste com os achados relatados por outros grupos^6^. Ao longo do estudo, os níveis de BNP e homocisteína não variaram. Embora os valores de PAM pré-diálise tenham sido ligeiramente mais elevados durante a hemodiafiltração em comparação aos dois períodos de hemodiálise, não houve diferenças na variabilidade da pressão arterial intradialítica. É possível que isso tenha ocorrido porque o regime de seis sessões semanais e a consequente redução do intervalo interdialítico são mais importantes para a estabilidade cardiovascular e a redução da morbidade do que a modalidade de diálise em si^3,5,29,30^.

Os níveis de hemoglobina pré-diálise foram mais elevados ao final do período de hemodiafiltração em comparação aos dois períodos de hemodiálise. Não foram observadas diferenças significativas nas doses semanais do agente estimulador da eritropoietina e da suplementação de ferro, nos índices de saturação da transferrina ou nos níveis de ferritina. Esses achados estão de acordo com os de estudos anteriores^7,31^ e possivelmente estão relacionados a uma redução na resistência aos agentes estimuladores da eritropoiese observada na hemodiafiltração, devido ao aumento da depuração de hepcidina^32,33^.

Considerando o perfil lipídico, os níveis de colesterol HDL foram significativamente mais elevados no final do período de SDHDF em comparação aos dois períodos de hemodiálise. Esse achado está em consonância com uma comparação anterior entre hemodiafiltração e hemodiálise de baixo fluxo^8^. Uma melhora na remoção de inibidores circulantes da lipoproteína lipase durante a hemodiafiltração pode explicar esse aprimoramento no perfil lipídico^8,34^.

Não foram observadas diferenças nos parâmetros osteometabólicos entre a hemodiafiltração curta diária e a hemodiálise curta diária. Este achado contrasta com os achados de diversos estudos anteriores, que demonstraram alguma melhora no controle do fosfato e/ou nos níveis de PTH pré-diálise após a realização de hemodiafiltração três vezes por semana, em comparação à hemodiálise feita com a mesma frequência^8,35,36^.

Tampouco foram observadas alterações nos marcadores inflamatórios ao longo do estudo. Este resultado difere dos resultados de outros trabalhos, que relataram uma melhora no perfil inflamatório após a hemodiafiltração em comparação à hemodiálise^37-39^. Não foram observadas diferenças nos escores dos componentes físico e mental do SF-12 entre as modalidades de tratamento avaliadas neste trabalho, semelhante ao relatado em um estudo publicado anteriormente^40^. Não houve diferenças observadas também nos escores totais de desconforto dos sintomas avaliados pelo ESAS-r: Renal. É possível que não apenas o regime diário de curta duração, mas também o uso de água ultrapura e dialisadores de alto fluxo altamente biocompatíveis durante todo o estudo tenham diminuído as possíveis vantagens da própria hemodiafiltração em relação à redução da inflamação e à melhora da qualidade de vida e dos sintomas.

A principal limitação do presente estudo foi o fato de ter sido realizado em um único centro, utilizando uma amostra de tamanho reduzido. São necessários estudos multicêntricos com um número maior de pacientes para confirmar os achados do nosso estudo.

## Conclusão

Em conclusão, nosso estudo revelou diversos achados importantes sobre os efeitos da hemodiafiltração online diária de curta duração em comparação à hemodiálise curta diária de alto fluxo. Observamos um aumento discreto, porém significativo, na pressão arterial média pré-diálise, na hemoglobina e nos níveis de colesterol de lipoproteína de alta densidade em pacientes submetidos à hemodiafiltração. Além disso, houve uma maior remoção de solutos de peso molecular médio durante a hemodiafiltração, o que pode contribuir para melhores desfechos para os pacientes. Esses resultados sugerem que as melhorias bioquímicas e hemodinâmicas associadas à hemodiafiltração podem ser benéficas. A longo prazo, a hemodiafiltração diária curta pode trazer mais benefícios para a população em diálise. Diante desses desfechos promissores, há um argumento convincente para a adoção da hemodiafiltração como padrão de tratamento em contextos de diálise frequente.

## Data Availability

Os dados que sustentam os achados deste estudo estão disponíveis mediante solicitação razoável ao autor correspondente.
